# Network-based analysis of key regulatory genes implicated in Type 2 Diabetes Mellitus and Recurrent Miscarriages in Turner Syndrome

**DOI:** 10.1038/s41598-021-90171-0

**Published:** 2021-05-21

**Authors:** Anam Farooqui, Alaa Alhazmi, Shafiul Haque, Naaila Tamkeen, Mahboubeh Mehmankhah, Safia Tazyeen, Sher Ali, Romana Ishrat

**Affiliations:** 1 Centre for Interdisciplinary Research in Basic Sciences, Jamia Millia Islamia, New Delhi, 110025 India; 2 Medical Laboratory Technology Department, Jazan University, Jazan, Saudi Arabia; 3 Research and Scientific Studies Unit, College of Nursing and Allied Health Sciences, Jazan University, Jazan, Saudi Arabia; 4 Department of Biosciences, Jamia Millia Islamia, New Delhi, 110025 India; 5 Department of Life Sciences, Sharda University, Greater Noida, 201310 India

**Keywords:** Computational biology and bioinformatics, Evolution, Genetics, Biomarkers

## Abstract

The information on the genotype–phenotype relationship in Turner Syndrome (TS) is inadequate because very few specific candidate genes are linked to its clinical features. We used the microarray data of TS to identify the key regulatory genes implicated with TS through a network approach. The causative factors of two common co-morbidities, Type 2 Diabetes Mellitus (T2DM) and Recurrent Miscarriages (RM), in the Turner population, are expected to be different from that of the general population. Through microarray analysis, we identified nine signature genes of T2DM and three signature genes of RM in TS. The power-law distribution analysis showed that the TS network carries scale-free hierarchical fractal attributes. Through local-community-paradigm (LCP) estimation we find that a strong LCP is also maintained which means that networks are dynamic and heterogeneous. We identified nine key regulators which serve as the backbone of the TS network. Furthermore, we recognized eight interologs functional in seven different organisms from lower to higher levels. Overall, these results offer few key regulators and essential genes that we envisage have potential as therapeutic targets for the TS in the future and the animal models studied here may prove useful in the validation of such targets.

## Introduction

The medical systems and scientists throughout the world are under an unprecedented challenge to meet the medical needs of much of the world’s population that are suffering from chromosomal anomalies. As there is no cure for such anomalies, early detection and specific interventions are likely to be the key aspect of economically efficient and high-quality healthcare systems. Turner Syndrome (TS), also known as monosomy of X, is one such chromosomal disorder where there is a complete or partial deletion of an X chromosome in all or some of the somatic cells^1,2^, usually due to sporadic chromosomal non-disjunction. TS is the only chromosome that represents haploinsufficiency compatible with life in less than 1% surviving pregnancy^3^. The reason why some TS fetuses are miscarried while others make it through pregnancy without many complications is still unknown. The most likely reason for the TS fetus’s survival may be the level of mosaicism that enables them to remain alive and develop. It has been reported that about 50% of TS patients have haplotype 45, X, while about 20–30% have mosaicism where 45, X cell line is accompanied by one or more other cell lines having a complete or structurally abnormal sex chromosome (X or Y)^4,5^. Some of the distinctive symptoms of TS include short height, webbed neck, low hairline at the back of the neck, low-set ears, markedly elevated levels of follicle-stimulating hormone (FSH), chronic otitis media (OM), lymphedema of extremities, small mandible, and multiple pigmented nevi^6^. TS is often accompanied by many other co-morbidities like autoimmune diseases (AD), hypothyroidism, kidney dysfunction, loss of ovarian function or other reproductive disorders, neurological or ophthalmological abnormalities, osteoporosis, diabetes mellitus (DM), dyslipidemia, recurrent miscarriages (RM), hypertension, and heart disease^7–12^. Since TS is non-heritable and no good animal model is available, this further complicates its status^13^. Therefore, the causal genes of TS co-morbidity are still unstated.

The prevailing theory of gene dosage imbalance due to the loss of an X chromosome is not enough to justify this burden of co-morbidity which has worsened the living circumstances of these patients. Two such acquired co-morbidities of TS which is the focus of our present work are Type 2 Diabetes Mellitus (T2DM) and Recurrent Miscarriages (RM). Even though T2DM and RM seem to be extremely frequent, there is a void of literature related to them.

Reports suggest that the prevalence of T2DM occurs at any age group in life. Some studies suggest that impaired beta-cell function or reduced insulin sensitivity may be the causal factor for T2DM in TS^14^. The latest clinical practice guidelines for TS states that glucose intolerance and T2DM prevalence in TS is 15–50% and 10%, respectively; nevertheless, the frequency of T1DM is yet to be determined^1^. It is expected that the factors that trigger T2DM in TS are different from that of the traditional risk factors of T2DM in the general population. It is currently believed that Xp haplotype gene deficiency may be the reason for the high incidence rate of T2DM in the TS population which may lead to impaired β-cell function^15^. Further, overexpression of some genes of Xq may worsen the condition. Whilst these factors may account for T2DM in TS, the exact reason and the key genes involved remain obscure.

Another distinctive feature of TS is ovarian failure which renders the TS women infertile in most cases. However, in a few of the TS cases i.e. 5–10%, spontaneous puberty occurs of them only 2–5% become pregnant spontaneously^16^. These pregnancies are at high risks and must be followed up carefully. Recurrent miscarriages are defined as two or more successive pregnancy losses before 22 weeks of gestation. It has been assessed that RM occurs in 0.5–5% of all reproductive-age women^17^, however, this rate is higher in turner's population^18^. Despite worthy studies that have ascribed the increased risk of miscarriage in TS patients to small uterine size and reduced endometrial thickness and receptivity^18,19^, little is known about it at the genomic level. Therefore, such genetic alterations leading to the enhanced risk of RM in TS women must be evaluated.

Many studies have shown that impaired glucose tolerance, diabetes mellitus and Insulin Resistance (IR) have somehow been responsible for adverse reproductive/pregnancy outcomes, including infertility and miscarriages^20,21^. However, there is little information and data to verify this claim. Since the data claims that in the general population, T2DM and RM may somehow be associated, we expect a similar scenario in the case of the TS population.

The molecular components of a human cell are functionally interdependent which means that a disease or a syndrome is a consequence of the perturbations of the complex intracellular and intercellular interactions. With the advancement in the field of Network biology, many potential disease genes and their better drug targets have been identified. The emerging tool of Network medicine has important applications to systematically explore drug targets, biomarker/key genes of the network through the identification of disease modules and pathways, thus providing improvements in the diagnosis, prognosis, and treatment of complex diseases^22–26^. In our study, the proposed network protocol not only provides a global analysis of the TS proteins but also presents a detailed view on specific proteins and their association with the specific co-morbidities i.e., T2DM and RM. The current research also sheds light on few key genes, pathways, and some interologs which can be targeted to offer better interventions for TS along with its associated co-morbidities.

## Results

**Figure 1:**
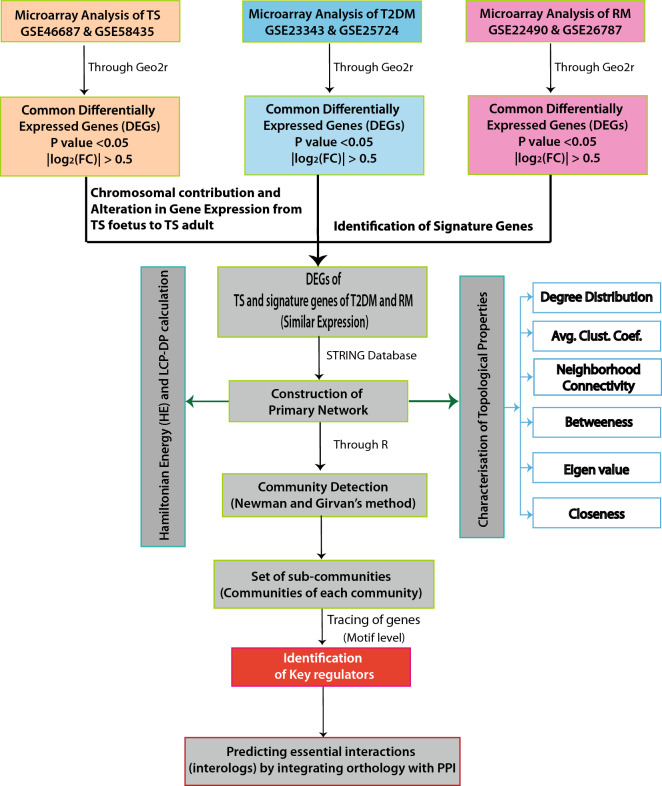
Illustration of the workflow of the integrative network-based approach of our study.

The workflow of the whole integrative network-based approach followed in this study is illustrated in Fig. 1. The figures in this study were drawn in Adobe Illustrator CS6.

### Differentially expressed genes (DEGs) of TS

The information regarding the datasets pertaining to the microarray series used in the study is listed in Table 1. The box plots for the normalized data sets are illustrated in Supplementary Figures S1−S3. Only those DEGs that surpassed the cut-off criteria of “P-value < 0.05” and “|logFC|≥ 0.5” in both the series of each category were considered as the significant DEGs. We did not use the adjusted p-value for multiple comparisons, rather we used the p-value cut-off < 0.05 to select the maximum number of DEGs of Turner Syndrome and then proceeded with the network construction. The DEGs of each dataset that surpassed the cut-off criteria are listed in Supplementary Table S1. A total of 355 genes were found to be differentially expressed in TS, of them 239 genes were upregulated, and 116 genes were downregulated (Supplementary Figure S4A). These genes were used to construct the PPI network to get the elaborated view in terms of protein–protein interactions in TS.

**Table 1:** Microarray datasets of (A) Turner Syndrome (B) Type 2 Diabetes Mellitus (C) Recurrent Miscarriage.

Geo accession	Platform	No. of probes	No. of samples (control/disease)	Sample type	Organism
**(A) Turner Syndrome**					
GSE46687	GPL570	54,675	36 samples (10/26)	peripheral blood mononuclear cells	Homo sapiens
GSE58435	GPL570	54,675	10 samples (5/5)	Second trimester amniotic fluid	Homo sapiens
**(B) Type 2 Diabetes Mellitus**					
GSE23343	GPL570	54,675	17 samples (7/10)	Liver tissue	Homo sapiens
GSE25724	GPL96	22,283	13 samples (7/6)	Pancreatic islets	Homo sapiens
**(C) Recurrent Miscarriage**					
GSE22490	GPL570	54,675	10 samples (6/4)	Placenta	Homo sapiens
GSE26787	GPL570	54,675	15 samples (5/10)	Endometrium	Homo sapiens

### Signature genes of T2DM and RM in TS

**Figure 2:**
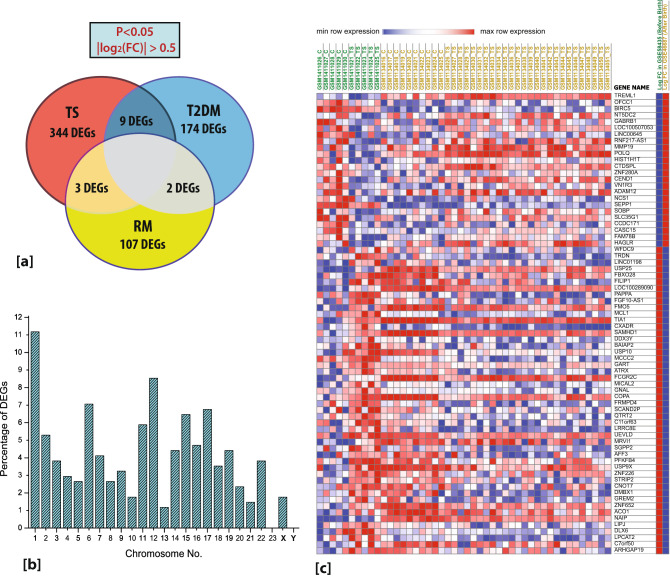
(**a**) Signature genes of T2DM and RM in TS. (**b**) The percentage of genes on each chromosome that was differentially expressed in TS at the significance threshold “P-value < 0.05” and “|logFC|≥ 0.5”. (**c**) The heat map showing the change in expression of the DEGs from TS fetus (GSE58435) to TS adult (GSE46687).

Here, we adopted an integrative approach for the meta-analysis of multiple gene expression profiles of TS and its comorbidities i.e., T2DM and RM, specifically. Using the same cut-off criteria, a total of 185 genes were found to be differentially expressed in T2DM, of them 138 genes were upregulated, and 47 genes were downregulated (Supplementary Figure S4B, Supplementary Table S2). And in the case of RM, 112 genes were found to be differentially expressed, 42 of them were upregulated, and 70 genes were downregulated (Supplementary Figure S4C, Supplementary Table S3). On overlapping these sets of DEGs, 9 genes were found to be common with TS and T2DM and 3 genes were common with TS and RM (Fig. 2a). We call them the “Signature Genes” that may be responsible for T2DM and RM in TS. These signature genes are listed in Table 2. The values of the fold change and p-value of the DEGs and the signature genes of TS, T2DM, and RM are listed in the Supplementary Tables.

**Table 2:** List of signature genes of T2DM and RM in TS.

S. no	Gene name	Chromosomal location	Status in TS	Status in T2DM
1	SLC29A2	11q13.2	Upregulated	Upregulated
2	THBS1	15q14	Upregulated	Upregulated
3	GPRC5B	16p12.3	Upregulated	Upregulated
4	CSHL1	17q23.3	Upregulated	Upregulated
5	ADAM22	7q21.12	Upregulated	Upregulated
6	IGHM	14q32.33	Upregulated	Upregulated
7	WIZ	19p13.12	Upregulated	Upregulated
8	IGHD	14q32.33	Upregulated	Upregulated
9	COX11	17q22	Downregulated	Downregulated

S. no	Gene name	Chromosomal location	Status in TS	Status in RM
1	ATXN7L1	7q22.3	Upregulated	Upregulated
2	UBE3B	12q24.11	Upregulated	Upregulated
3	FANCM	14q21.2	Downregulated	Downregulated

### Association between T2DM and RM in TS through pathway analysis

The pathway enrichment and functional enrichment was analyzed for all the DEGs of TS along with their interacting partner. We found that the DEGs were significantly enriched in many biological, cellular, and molecular functions as well as some pathways (Table 3). Most of these genes are enriched in protein binding, ATP binding, RNA binding, and translational processes. Supplementary Figure S5 illustrates the involvement of different pathways in TS. Furthermore, in the current study, we evaluated the association between RM and T2DM in TS. For this, we selected the signature genes of RM and T2DM in TS along with their interacting partners in the TS network. These genes are **THBS1** (MIM: 188,060), NFATC1 (MIM: 600,489), **GPRC5B** (MIM: 605,948), FANCL (MIM: 608,111), **WIZ** (GenBank: NM_021241.2), JRK (MIM: 603,210), XAB2 (MIM: 610,850), **CSHL1** (MIM: 603,515), LSM5 (MIM: 607,285), **COX11** (MIM: 603,648), RPL3L (MIM: 617,416), **ADAM22** (MIM: 603,709), LGI1 (MIM: 604,619), **SLC29A2** (MIM: 602,110), **IGHM** (MIM: 147,020), **IGHD** (MIM: 147,170), **ATXN7L1** (GenBank: NM_020725.1), SF3B3 (MIM: 605,592), **UBE3B** (MIM: 608,047), SLC9A9 (MIM: 608,396), **FANCM** (MIM: 609,644), SMARCAL1 (MIM: 606,622), MUS81 (MIM: 606,591), TIPIN (MIM: 610,716), RECQL5 (MIM: 603,781), MLH1 (MIM: 120,436), RMI1 (MIM: 610,404), RMI2 (MIM: 612,426), ERCC4 (MIM: 133,520), and RAD52 (MIM: 600,392). The highlighted genes in bold are signature genes. It was found that ten of these genes (ERCC4, RMI1, FANCM, FANCL, RAD52, XAB2, MUS81, TIPIN, RMI2, and MLH1) are involved in **DNA repair** of which four of them i.e., ERCC4, FANCM, FANCL, and MUS81 are specifically involved in **Fanconi anemia pathway** which is also a type of DNA repair pathway. We expect that these two pathways are important as they have common genes of RM and T2DM predicted in the study.

**Table 3:** The gene ontology and pathway enrichment of DEGs of TS, signature genes of T2DM and RM and their interacting partners.

Go term	Total no. of genes involved from the list	Seed genes present	P-value
**Molecular function**			
Protein binding	403	ZNF703, ZNF24, ZKSCAN3, ZFYVE27, ZFHX3, ZBTB17, ZBTB10, YTHDC2, XPO5, WWOX, WRNIP1, **WIZ**, WBP2, VPS37B, USP22, UBE2Z, TYRP1, TTC23, TSPAN2, TPM1, TOPORS, TOB2, TNXB, TMEM8A, TMEM67, THY1, **THBS1**, TBX5, TAF1C, SYNE2, SYNCRIP, STMN2, STK16, STAT2, STAT1, SRGAP2, SRC, SPON1, SPG7, SOX2, SOX13, SOCS3, SNRPN, SMYD2, SLC9A9, SFI1, SETDB1, SEPT6, RYK, RPL31, RPH3AL, RAB3IP, PXN, ***PTPN22***, PPP6R2, ***POU2F1***, POSTN, POGK, PMEPA1, PKHD1, PKD1, PHF1, PEX3, PDLIM5, PDE4DIP, PCNX4, PBXIP1, PBX2, PAPOLA, OLFM1, OCIAD1, NR4A1, NPC1, NIPSNAP1, NFATC3, NFATC1, NF2, NEK8, MKL1, MAPT, MAPK1, MAGEC2, LRR1, LIFR, LGALS3, ***LCP2***, LAPTM4B, KRR1, KRAS, KMT2A, KIF1C, KCNJ15, JRK, ITPR3, ITIH4, IL23A, IL1R2, IKZF3, **IGHM**, IFT27, HSPA1L, HIPK2, HIPK1, HFE, HEXIM1, HBB, GUCY1A3, GSE1, GOPC, GNAS, GADD45B, FOXN3, FOXK1, FLT1, FKBP8, FGFR1OP2, FBLIM1, **FANCM**, FANCC, ***FAM20A***, FAM161A, FAF1, ETV6, ETS2, ENO2, ***ENAM***, EDC3, DYRK1A, DTNA, DOT1L, DGKZ, DCAF8, CXCR5, CUL9, CRTC2, CNOT2, CEP152, CELF1, CEACAM1, CDK15, CDC34, CASP2, CASK, CARS, CACNG2, BICD1, BCL6, BCL2L1, BAZ2A, **ATXN7L1**, ATF6B, ASXL1, ARID4B, ARHGAP22, APLP2, AP5Z1, ANKRD44, ALOX5, AGO4, AGER, ADRB1, **ADAM22**, ACHE, ABHD1	1.3E-21
poly(A) RNA binding	103	YTHDC2, XPO5, SYNCRIP, SAMD4B, RPL31, PURB, OASL, LGALS3, KRR1, KIF1C, FNDC3B, FBRSL1, CELF1	6.8E-21
ATP binding	82	YTHDC2, YME1L1, WRNIP1, UBE2Z, TRIB2, STK16, SRC, SPG7, SCYL1, RYK, RPS6KA6, RECQL5, PKDCC, PFKP, PDXK, PDK4, PCCB, PAPOLA, OASL, NEK8, MAST4, MAPK1, KRAS, KIF1C, KALRN, HSPA1L, HIPK2, HIPK1, FLT1, **FANCM**, EPHA8, EIF2AK4, EARS2, DYRK1A, DTYMK, DNAH1, DGKZ, CUL9, CDK15, CDC34, CASK, CARS, ATP9B, ABCC1	8.8E-6
RNA binding	71	XPO5, SYNCRIP, SNRPN, SAMD4B, RPL31, PAPOLA, EARS2, CELF1, BAZ2A	1.3E-22
structural constituent of ribosome	48	IKZF3, IFIT3, EDC3, CASP8, SEPT7, IL12B, CASP2, AKT1, ABCD1, EPHA4, APAF1, EED, ACTN2, APLP2, DYRK1A, WRNIP1, CREB1, PRPF3, FKBP8, VWA1, RAF1, SNRNP200, TP53, KMT2A, XIAP, **THBS1**, AGER, MAPK1, CSK, FYN, BAK1, AMELX, PLK4, JUN, STAT1, sSTAT2, STAT3, FN1, BRAF, S100B, VEGFA, RAD52, BCL6, BCL2, MDM2, BAX, FAS, GRB2, BCL2L1	9.9E-25
**Cellular component**			
Nucleus	252	ZNF782, ZNF703, ZNF684, ZNF24, ZKSCAN3, ZFHX3, YTHDC2, XPO5, WWOX, WRNIP1, **WIZ**, **UBE3B**, TXNDC2, TRIB2, TOPORS, TOB2, TMEM57, TBX5, SYNE2, SYNCRIP, STC1, STAT2, STAT1, SRGAP2, SRC, SOX2, SOX13, SMYD2, SETDB1, SCYL1, SBF1, SAMD4B, RYK, RPS6KA6, RECQL5, RAB3IP, PURB, ***PTPN22***, ***POU2F1***, POGK, PKD1, PHF1, PFKP, PERM1, PDXK, PDE4DIP, PBXIP1, PBX2, PAPOLA, NR4A1, NFATC3, NFATC1, NF2, MTF2, MOB2, MKL1, MAPK1, MAGEC2, LMO7, LGALS3, KRR1, KMT2A, KLF16, KDM4B, KANSL3, JRK, IKZF3, HIVEP2, HIPK2, HIPK1, HEXIM1, HES2, **GPRC5B**, GNAS, GADD45B, FOXN3, FOXK1, FANCC, FAM71B, FAF1, ETV6, ETS2, EFCAB13, DYRK1A, DTYMK, DOT1L, DGKZ, DCAF8, CRTC2, CNOT2, CELF1, CDC34, CASP2, CAMTA1, BTBD7, BCL6, BAZ2A, ATF6B, ARID4B, ARHGAP22, ARG1, APLP2, AP5Z1, AGO4, ACHE	2.2E-12
Cytoplasm	230	ZNF703, ZKSCAN3, ZFHX3, XPO5, WWOX, VPS37B, **UBE3B**, UBE2Z, TXNDC2, TRIB2, TOB2, TBX5, TBC1D32, SYNE2, STMN2, STK16, STC1, STAT2, STAT1, SRGAP2, SRC, SOX2, SOCS3, SNRPN, SMYD2, SETDB1, SCYL1, SAMD4B, RYK, RPS6KA6, RPH3AL, RNF213, RGS5, RECQL5, PXN, ***PTPN22***, PPP6R2, POSTN, PKHD1, PKD1, PHF1, PFKP, PERM1, PDXK, PDLIM5, PDE4DIP, PAPOLA, OASL, NR4A1, NFATC3, NFATC1, NF2, NEK8, MYADML2, MTF2, MOB2, MMP28, MKL1, MAST4, MAPT, MAPK1, MAGEC2, MAFIP, LST1, LMO7, LGALS3, KRR1, KRAS, KMT2A, KDM4B, JRK, ITPR3, IL1R2, IKZF3, HIPK2, HIPK1, HEXIM1, GOPC, GNB1L, GNAS, GADD45B, FGD6, FANCC, FAM161A, ETV6, ETS2, EML4, EFCAB13, EARS2, DTNA, DNAH1, DGKZ, DCAF8, CUL9, CRTC2, CNTLN, CNOT2, CELF1, CDH3, CDC34, CD96, CASP2, CASK, CARS, CAMTA1, BCL2L1, BAZ2A, ARID4B, ARG1, AQP4, AP5Z1, ANKRD13D, AKAP10, AGO4	1.1E-8
Cytosol	211	XPO5, WWOX, TPM1, THY1, TAT, STAT2, STAT1, SRGAP2, SRC, SOX2, SOCS3, SMYD2, SFI1, RPS6KA6, RPL31, RNF213, RGS1, RAB3IP, PXN, PFKP, PEX3, PDXK, PDLIM5, PCCB, PBXIP1, OASL, NFATC3, NFATC1, MTHFR, MKL1, MAPT, MAPK1, ***LCP2***, KRAS, KALRN, HSPA1L, HBG2, HBG1, HBB, HAL, GNAS, FGFR1OP2, FERMT1, FBLIM1, FANCC, FAF1, ENO2, EDC3, DTYMK, DENND1C, CNOT2, CNBD2, CEP152, CASP2, CASK, CARS, BICD1, BET1L, BCL2L1, ARHGAP22, ARG1, ALOX5, AKAP10, AGO4	1.1E-25
Nucleoplasm	186	ZFHX3, ZBTB17, ZBTB10, YME1L1, XPO5, UBE2Z, TBX5, TAF1C, SYNE2, SYNCRIP, STAT2, STAT1, SRGAP2, SOX2, SOX13, SMYD2, SETDB1, RPS6KA6, RECQL5, PXN, ***POU2F1***, PMEPA1, PHF1, PEX3, PDXK, PAPOLA, NR4A1, NFATC3, NFATC1, MTF2, MKL1, MAPK1, KRR1, KMT2A, KDM4B, KANSL3, ITPR3, HSPA1L, HIVEP2, HIPK2, HIPK1, HEXIM1, **FANCM**, FANCC, EZH1, ETS2, EFCAB13, DYRK1A, DOT1L, DGKZ, CRTC2, CEP152, CELF1, CDC34, BOD1L1, BCL6, BAZ2A, ARID4B, AP5Z1	1.5E-24
Membrane	139	YME1L1, WRNIP1, TM7SF2, SYNCRIP, STMN2, STK16, SLC1A6, SLC12A9, SCYL1, RYK, RPL31, RNF213, PRSS12, PFKP, PEX3, PDLIM5, PCSK6, PCDHGC3, OCIAD1, OASL, NPC1, NNT, NF2, MUC4, LST1, LGALS3, LAPTM4B, KRR1, KRAS, KIAA2013, KCNJ15, ITPR3, GOPC, GNAS, GGCX, GALNT1, FKBP8, EML4, EDC3, CNOT2, CELF1, CEACAM1, CDH3, CASP2, BICD1, BET1L, BCL2L1, B4GALT1, AQP4, APLP2, ALG12, AGO4, ACHE, ABCC1	1.6E-15
**Biological process**			
rRNA processing	62	RPL31, KRR1	1.4E-39
positive regulation of transcription from RNA polymerase II promoter	54	WWOX, WBP2, TBX5, STK16, STAT1, SOX2, PKD1, PBX2, NR4A1, NFATC3, NFATC1, MTF2, MKL1, KMT2A, IL23A, IKZF3, HIPK2, EZH1, ETV6, ETS2, DOT1L, CRTC2, CASK, CAMTA1, ASXL1, ARID4B	5.0E-4
Translational	51	RPL31	1.4E-24
translational initiation	49	RPL31	5.1E-36
nuclear-transcribed mRNA catabolic process, nonsense-mediated decay	47	RPL31	8.1E-37
**Reactome pathway**			
Formation of a pool of free 40S subunits	53	RPL31	1.11E-16
Nonsense Mediated Decay (NMD) independent of the Exon Junction Complex (EJC)	49	RPL31	1.11E-16
GTP hydrolysis and joining of the 60S ribosomal subunit	53	RPL31	1.11E-16
L13a-mediated translational silencing of Ceruloplasmin expression	53	RPL31	1.11E-16
SRP-dependent co-translational protein targeting to membrane	52	SEC11C, RPL31	1.11E-16
**KEGG pathway**			
hsa03010: Ribosome	48	RPL31	2.7E-27
hsa03040: Spliceosome	32	HSPA1L	6.2E-13
hsa05161: Hepatitis B	32	STAT2, STAT1, SRC, NFATC3, NFATC1, MAPK1, KRAS, ATF6B	7.1E-12
hsa03460: Fanconi anemia pathway	20	**FANCM**, FANCC	5.7E-12
hsa04360: Axon guidance	28	SRGAP2, NFATC3, MAPK1, KRAS, EPHA8, ***EFNB3***	2.0E-10

Key genes are bold and italicised and signature genes are bold.

### Chromosomal contribution of DEGs

To study the genomic imbalance, we studied the percentage contribution of the DEGs of TS across the chromosomes. It was observed that all the chromosomes had altered gene expression except chromosomes 23 and Y. It ranged from 1.17% (chromosome 13) to 11.17% (chromosome 1) (Fig. 2b). Chromosome X contributes to 1.76% of DEGs. Chromosome 1 had the highest proportion of significantly altered genes in the study. Thus, it was observed that TS phenotype is the result of global genomic imbalance, rather than the genes of individual X chromosome alone^27^. One could therefore explain the TS phenotype only by the additive effect of genes based on different loci.

### Change in expression of genes in TS fetus and TS adult

Genes identified as expressed in fetal tissues may provide clues to developmental processes and are a candidate set for further analysis in disease studies. There are few genes whose expression changes on transformation from fetus to adult contributing to different developmental processes of that individual. As mentioned in Table 1, GSE58435 signifies the expression of genes in TS fetus while GSE46687 signifies the expression of genes in TS adult. We represented the transformation from TS fetus to TS adult in the form of a heat map (Fig. 2c) which was constructed using Morpheus online tool. In a comparison of genes expressed in fetal vs. adult turner patients, we identified 72 genes whose expressions are altered from fetus to adult in TS (P-value < 0.05 and |logFC|≥ 1). Of these 72 genes, 24 genes are downregulated in fetal turner patients and upregulated in adult turner patients while 48 genes are upregulated in fetal turner patients and downregulated in adult turner patients. These genes are of interest as candidate genes because their expression levels are just the opposite in TS individuals (fetus vs adult) in comparison to healthy controls. Therefore, it is expected that when these genes are activated in adult or fetal tissues in an altered fashion, this may hinder the developmental processes. The names of these genes are listed in Supplementary Table S4. These fluctuations of gene expressions from fetus to adult may contribute to the phenotypic features of the TS. The study presented here is only a subset of the types of information that these data sets can yield. Although the role of these 72 genes in TS is still unclear, our results elucidate a new aspect of TS which is also crucial for understanding its etiology which requires additional future analyses to provide further insights.

### Turner Syndrome network follows hierarchical scale-free features

The PPI network of TS was constructed with the DEGs of TS including the DEGs of T2DM and RM that were common with TS (signature genes). So, a total of 355 genes that were differentially expressed were used to construct the PPI network of TS. Our goal was to get a network that carries most of the signature genes in the same network with a condition that our network’s clustering coefficient must be greater than 0.5. The clustering coefficient being greater than 0.5 signifies that the network and its genes are finely clustered together. In our study, we tried different scenarios by changing the number of nodes in the first shell and second shell as mentioned below in Table 4.

**Table 4:** Parameters of network construction (confidence level 0.4).

S. no	No of nodes in 1st shell	No of nodes in 2nd shell	Total no of nodes and edges in final network (n,e)	Clustering coefficient	No of signature genes present in final network
1	250	50	(561, 11,230)	0.523	8 out of 12
2	300	100	(672, 16,353)	0.532	9 out of 12
3	500	100	(887, 24,388)	0.534	9 out of 12
4	400	50	(723, 17,556)	0.539	8 out of 12
5	400	100	(775, 20,357)	0.544	9 out of 12

We found that most numbers of signature genes are incorporated in the 2nd, 3rd, and 5th cases. We, however, selected the 5th case i.e., 400 nodes in the 1st shell and 100 nodes in the 2nd shell as we get the best clustering coefficient here. Therefore, we proceeded with this case.

**Figure 3:**
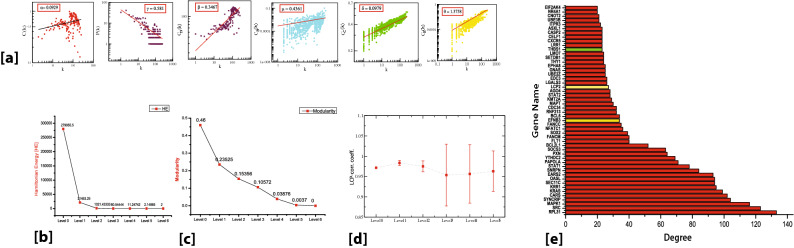
(**a**) The behaviours of degree distributions (P(k)), clustering coefficient (C(k)), neighborhood connectivity (C_N_(k)), betweenness (C_B_(k)), closeness (C_C_(k)) and eigen-vector (C_E_(k)) measurements as a function of degree k for TS network. (**b**) Corresponding Hamiltonian Energy (HE) as a function of levels of organization. (**c**) Corresponding modularity Q_N_ as a function of levels of organization (**d**) Variation in the calculated average LCP-corr for TS network as a function of network-level. (**e**) Characterization of top fifty leading hubs of the network by degrees. EFNB3 and LCP2 are the key regulators denoted by yellow color and THBS1 are the signature gene of T2DM denoted by green color.

Of 355 DEGs, only 271 genes made it to the main network. We call these genes seed genes. The main constructed network consisted of 775 nodes and 20,357 edges. The nodes here are the proteins and the edges are the interaction between these proteins. Protein–protein interactions (PPIs) can be conveniently represented as networks, allowing the use of graph theory for their study. Studying the topological properties of the TS network may reveal patterns associated with TS in humans. The topological properties used here are the probability of degree distribution P(k), clustering coefficient C(k), and neighborhood connectivity C_N_(k). They characterize the structural and organizational features of the TS network. It was observed that these topological properties obey power-law behavior as a function of degree k (Fig. 3a). The power law of the datasets of the topological variables of the TS network is fitted and verified following a standard statistical fitting procedure proposed by Clauset et al.^28^. The values of the exponents are attained from the power-law fittings. The summarised results for the complete network are as follows,

1 $$ \left[ {\begin{array}{*{20}c} P \\ C \\ {C_{N} } \\ \end{array} } \right]\sim \left[ {\begin{array}{*{20}c} {k^{ - \gamma } } \\ {k^{ + \alpha } } \\ {k^{ + \beta } } \\ \end{array} } \right] \to \left[ {\begin{array}{*{20}c} {0.581} \\ {0.0929} \\ {0.3467} \\ \end{array} } \right] $$

These values suggest that the TS network follows a weak hierarchy. The value of $\gamma$ signifies that the number of nodes increases with the advancement of disease as a power of 0.581, thus, giving us the idea of the TS network being hierarchical as it shows the presence of modules in our clustering experiment. The graphical representation of degree distribution P(k) shows that the network is dominated by lower degree hubs than that of higher degree hubs. This signifies that the network follows the power law and thus the hierarchical, scale-free, and has fractal attributes. The positive value of β suggests that the network carries the assortive mixing specifying that a large cluster of degree nodes (formation of the rich club) regulates the TS network.

The centrality measurements correspond to the flow of information in the network and predict the influential candidates in the network that play important role in the flow of information in the network. Two such measures are betweenness centrality C_B_(k) and closeness centrality C_C_(k). The eigenvector centrality C_E_(k) depicts the efficacy of the spreading (receiving) power of information of nodes from the network. These properties obey power-law behaviors as follows,

2 $$ \left[ {\begin{array}{*{20}c} {C_{B} } \\ {C_{C} } \\ {C_{E} } \\ \end{array} } \right]\sim \left[ {\begin{array}{*{20}c} {k^{\mu } } \\ {k^{\delta } } \\ {k^{\theta } } \\ \end{array} } \right] \to \left[ {\begin{array}{*{20}c} {0.4361} \\ {0.0979} \\ {1.376} \\ \end{array} } \right] $$

Following the procedure of Clauset et al*.* (2009), these three centrality measurements are again verified and confirmed for their statistical power-law fits. We found that only a few higher degree nodes have large centrality values which means that most of the influencing hubs that can control the network are few. Thus, the TS network is predominated by the low degree nodes (genes/proteins). It is these low-degree nodes that control the working and organization of the network. However, some of the leading hubs that are scarcely distributed might show significant involvement in regulating as well as stabilizing the network. Here, the positive values of these centrality measurements show that the network exhibits hierarchical scale-free or fractal features.

Thus, the overall topological properties of the TS network show that it self-organizes into a scale-free fractal state and is composed of successive interconnected communities which means the network has hierarchical organization.

### Validation of the biological significance of TS network

A network that differs significantly from a random network could be viewed as containing relevant information, assuming that the comparison with the random network is meaningful. Construction of the null model will allow us to assess the significance of the TS network features. A null model consists of one network (or a set of networks) that matches a graph under study in some structural aspects while being random in all other characteristics. In our study, we constructed two different null random networks of the same size and did the comparative analysis (Table 5).

**Table 5:** Comparative analysis of TS PPI network with random networks of the same size.

Properties	Main turner syndrome network	Degree preserved random network of TS	Random network (Erdos Renyi algorithm)
Nodes	775	775	775
Edges	20,357	20,357	20,357
Clustering co-efficient	0.544	0.294	0.068
Network diameter	7	5	3
Network radius	4	3	3
Exponent of average clustering co-efficient	0.092	− 0.091	− 0.0645
Exponent of degree distribution	− 0.581	− 0.581	0.125
Exponent of neighbourhood connectivity	0.346	− 0.0463	− 0.00934
Exponent of betweenness centrality	0.436	2.1	2.029
Exponent of closeness centrality	0.0979	0.092	0.084
Exponent of Eigen vector centrality	1.357	0.915	1.016

We can see that the clustering coefficient and the diameter of the random networks drastically decrease in comparison to the TS network. The exponents of other topological properties are also different and do not follow the scale-free fractal attributes. The graphical representation of topological properties of TS network and random networks as null models is illustrated in Supplementary Figure S6.

Through this comparative analysis, we found that the TS network constructed from the differentially expressed genes of TS is exclusively associated with TS and is not by chance and is biologically significant.

### Identification of key regulators and properties

To identify the systematic arrangements and modular structure of the TS network at their various levels of the organization, we followed Newman and Girvan’s standard community finding algorithm^29^. It was found that the TS network is hierarchically organized through six different levels. As one moves from top to down level of organization, the corresponding Hamiltonian Energy (HE) and modularity Q_N_ as a function of levels of the organization are found to be decreased (Fig. 3b,c, respectively).

**Figure 4:**
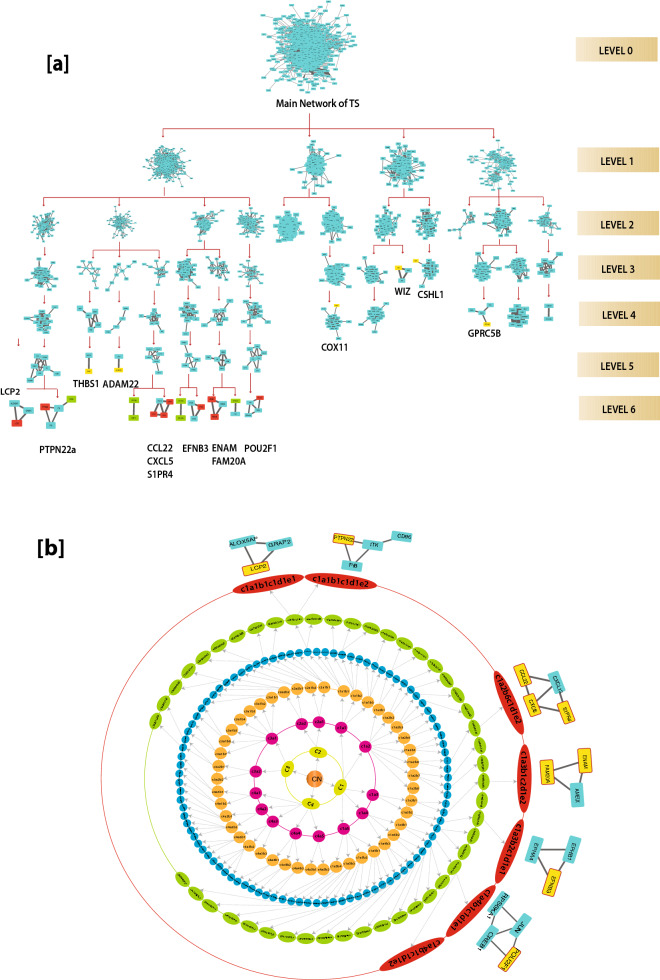
(**a**) Network/modules/sub-modules at different levels which accommodate leading hubs and key regulators. (**b**) Organization of the modules/sub-modules of the TS network.

The proteins that are deeply rooted from top to bottom of the network where the network cannot be further divided into sub-community and form motif are said to be the key regulators of the network which serve as the backbone of the network organization^30^. We identified nine key regulators LCP2, PTPN22, CCL22, CXCL5, S1PR4, POU2F1, FAM20A, ENAM, and EFNB3 (Fig. 4a) in the TS network. Surprisingly, none of these KR genes fall among the categories of the top ten leading hubs. However, two of the key regulators LCP2 and EFNB3 were among the top 50 high degree hubs (Fig. 3e). Thus, we can say that it is not necessary for these KRs to be the large leading hubs in the network, however, their popularities are randomly changed at various levels of organization (Fig. 4a,b). Since the network qualifies hierarchical characteristics, the elimination of the leading hubs will not cause its breakdown. But it is expected that these KRs, if eliminated, may cause maximum local and global perturbations, especially at a deeper level of organization. These perturbations may reach out to the deeper levels of organization causing the topological change in the network^30^.

Few more proteins of the seed genes i.e., CD96, CXCR5, IKZF3, SPON1, KALRN, and BTBD7 reached the sixth level but did not form the motif. Thus, they cannot be considered as the KRs. All the KRs maintained a low profile/popularity thereby regulating the network till the bottom level of the organization. None of the signature genes of T2DM and RM in TS reached the motif level, however, THBS1 and ADAM22 supported the network reached till the 5th level. THBS1 was among the top 50 high-degree hub genes. These KRs may propagate signals from top to bottom levels and vice versa of the network to maintain network stability and inherent properties. These key regulators are deeply rooted in the network, they serve as the backbone of the network for any network activities and regulations and could be a possible target gene for this disease control mechanisms.

Since these identified key regulators and the signature genes of TS are expected to play an important role in TS, we further explored them by searching the possible microRNAs that could regulate them. We used MIENTURNET tool^31^ which is an interactive web-based tool for microRNA-target enrichment analysis. The Supplementary Table S5 presents the MIENTURNET enrichment results of miRTarBase which gives the most up-to-date results for validated interactions. We used the p-value cut-off of < 0.05 to get the list of significant microRNAs responsible for the regulation of these key regulators and signature genes of TS.

**Figure 5:**
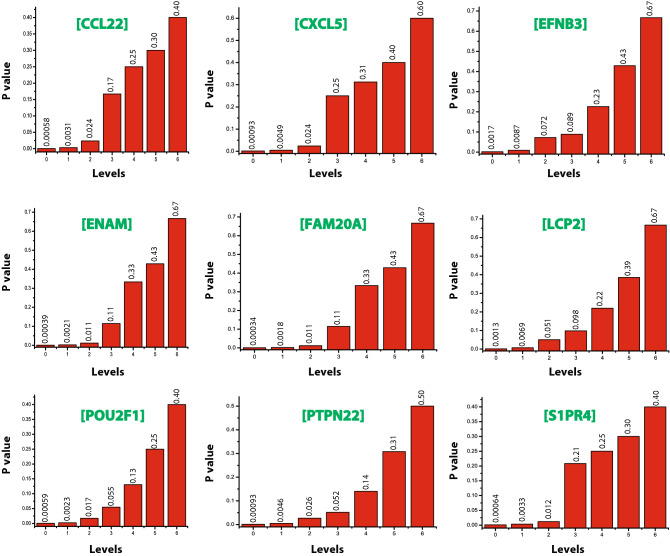
The probability distribution of the KRs as a function of the level.

The graphical representation of probability $P_{x} \left( {y^{l} } \right)$ of all the key regulatory genes show an increase in P_x_ from level 0 till 6th level (Fig. 5). This means the regulating ability of each key gene becomes more important and significant at the deeper level of organization.

### Evidence of self-organization: local-community-paradigm (LCP) approach

**Figure 6:**
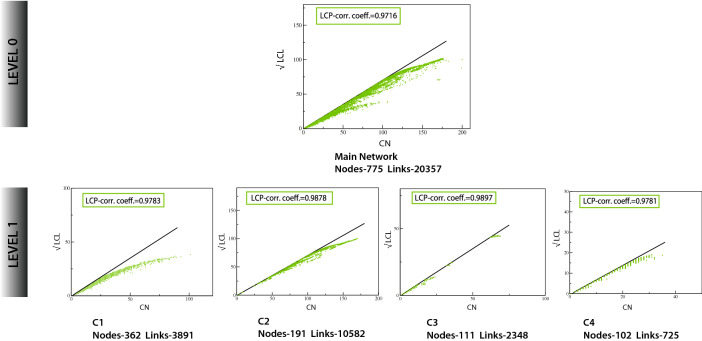
LCP-DP plots of main TS network (Level 0) and modules/sub-modules at the first level of organization of TS network.

The TS network was analyzed to assess the maintenance of its self-organization at different levels of its organization using the LCP technique. We calculated the LCP-correlation of all the communities/sub-communities through six different levels of its organization. The modules/communities having zero LCP-correlation were excluded in average. It was found that the average values of LCP-correlation at each level are greater than 0.95 and the values do not change with the error bar (Fig. 3d). The LCP-decomposition-plot (LCP-DP) for the main TS network (Level 0) and its sub-modules (4 sub-modules at Level 1) are shown in Fig. 6. Based on the nodes and their links of each network and its sub-modules we can conclude that the TS network and its sub-modules are more strongly characterized by small-local communities and are compact. This shows that the network is self-organized and compact with efficient information processing. The TS network represents a strong LCP network which lets us conclude that the network is dynamic and heterogeneous which enables network evolution and reorganization. Such architecture assists quick delivery of information across networks both locally and globally.

### Status of essential interactions of TS in non-human systems by integrating the Orthology with PPI

We examined the top hundred genes in each category i.e., degree distribution, betweenness centrality, closeness centrality, and eigenvector centrality. Eight such genes were identified which were common to these categories of Degree distribution and centralities and were also found to be the interacting partners of the seed genes in the TS network.

Besides, we identified 9 key regulators in the present study. Based on the assumption that the genes coding for the interacting proteins of disease-causing genes are putative, we also included the interacting partners of the key regulators of TS in the list (Supplementary Table S6).

Monosomy X, commonly called Turner Syndrome is not only limited to humans but such cases have also been reported in many other animal species^32^. In view of the facts that essential proteins evolve much slower than non-essential proteins^33^, we identified the orthologous counterparts of the proteins (Supplementary Table S6) in seven different organisms namely, *Mus musculus* (mice), *Rattus norvegicus* (Rat), *Felis catus* (domestic cat), *Ovis aries* (sheep), *Macaca mulatta* (rhesus macaque), *Gorilla Gorilla* (Gorilla), and *Homo sapiens* (Human) in our study.

Animal models are important in generating gain- and loss-of-function mutations of a syndrome/disease and have produced significant insights. In the case of TS, however, no animal models have been generated that exactly models it. In this study, we analyzed the status of essential interactions of TS in non-human systems by integrating the orthology with PPI. If two proteins physically interact in one species and they have orthologous counterparts in another species, it is likely that their orthologs interact in that species too. Such conserved interactions are called interologs which are of significant value in comparative genomics.

**Figure 7:**
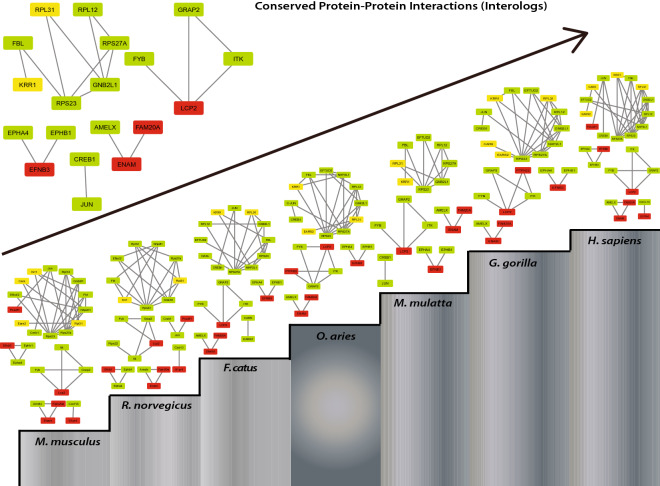
Interologs in the network from lower to higher organisms. Nodes in yellow are seed genes and nodes in red are seed genes that are the key regulators. Nodes in green are the interacting partners of seed genes.

So, the conserved interactions (interologs) were analyzed in these organisms (mentioned above). It was observed that only 18 protein–protein interactions involving 3 motifs (Table 6, seed genes highlighted in bold, Fig. 7) remained conserved in all organisms. Of these 18 interologs, 10 of them include the interaction of seed genes or key regulators with their neighbor which emphasizes their essential role in a living system. Their loss or gain of function may somehow affect the physiology of an individual which may result in the loss of an essential function. Therefore, apart from the identified key regulatory genes, we expect that these predicted interologs too might play a major role in the pathophysiology of TS. It is a matter of research for further insights. The animal models studied here might prove to be useful in illuminating the biological functions of these genes and the pathophysiology of TS associated with these genes. Clearly, this study does not conclude that these non-human animals are complete models for Turner syndrome as TS involves many genes. However, this is a powerful approach that can be used to select an appropriate model to study human disease.

**Table 6:** Essential PPI interactions in TS network.

S. no	Conserved interactions (interologs)	Genes forming motifs (conserved motifs)
1	ITK-GRAP2	ITK, GRAP2, LCP2
2	ITK-**LCP2**	
3	GRAP2-**LCP2**	
4	**LCP2**-FYB	RPL12, RPS27A, GNB2L1
5	**EFNB3**-EPHB1	
6	**EFNB3**-EPHA4	
7	**ENAM-FAM20A**	RPS23, RPS27A, GNB2L1
8	**ENAM**-AMELX	
9	**KRR1**-FBL	
10	FBL-RPS23	RPS23, RPL31, GNB2L1
11	RPS23-GNB2L1	
12	RPS23-**RPL31**	
13	GNB2L1-**RPL31**	
14	RPS23-RPS27A	
15	RPS27A-GNB2L1	
16	RPL12-GNB2L1	
17	RPL12-RPS27A	
18	JUN-CREB

Thus, such types of studies may identify gene targets for drug therapy of these individual pathologies in the general population and the animal models generated may prove useful in the validation of such targets.

## Discussion

TS is a consequence of a partial or total loss of the X chromosome which results in the onset of highly variable clinical features. Surprisingly, our knowledge of genotype–phenotype relations in TS is rather inadequate where very few specific candidate genes are linked to its clinical features. In this study, we used an integrative network-based approach to extract the information from the microarray datasets of TS. The study presented here is only a subset of the types of information that these data sets can yield, which requires additional future analyses to provide further insights.

It is expected that the causative factors of T2DM and RM in TS are different from that of traditional risk factors in the general population. We found out nine genes of T2DM namely, SLC29A2, THBS1, GPRC5B, CSHL1, ADAM22, IGHM, WIZ, IGHD, and COX11, and three genes of RM namely, ATXN7L1, UBE3B, and FANCM in TS. We call these genes as “signature” genes of T2DM and RM in TS. Previously reported studies were found to show the involvement of these genes in these conditions. The **SLC29A2 gene** which encodes the protein ENT2 (Equilibrative Nucleoside Transporter 2) has been proved sensitive to dysregulation in diabetes and acts as a target of insulin signaling^34^. It is known that dysfunction of pancreatic β-cell plays a critical role in the development of T2DM. In one of the studies, thrombospondin 1 (**THBS1**) has been found to play a crucial role in β-cell survival during lipotoxic stress in rat, mouse, and human models which suggests this to be an interesting therapeutic target to prevent oxidative stress in T2DM^35^. In another study, it was proposed that with the increase in expression of **GPRC5B** (G Protein-Coupled Receptor Class C Group 5 Member B) there is a reduction in insulin secretion and β-cell viability in T2DM^36^. Thus, GPRC5B too might prove to be a novel target for the prevention of T2DM. The human GH/CSH genes (**CSHL1** being one of them) regulates growth and are involved in fetal and adult glucose metabolism. This could act as a good target for gestational diabetes and diseases related to insulin resistance^37,38^. **ADAM22** (ADAM metallopeptidase domain 22) was found to be a potential target in insulin-resistant (IR) subjects, identified through an integrative miRNA–mRNA microarray and network approach in adipose tissue of IR and insulin-sensitive (IS) individuals^39^. A decreased expression of OXPHOS (oxidative phosphorylation) genes which included **COX11** (Cytochrome c oxidase assembly protein COX11) from pancreatic islets of T2DM patients was found in one of the studies which may lead to impaired insulin secretion^40^. Similarly, **FANCM**, identified to be the signature gene of RM in the present study is a DNA-damage response gene. FANCM protein was better expressed in pachytene cells where meiotic recombination occurs^41^. Therefore, any mutation or structural change in **FANCM** is expected to provoke meiotic defects resulting in DNA damage. The accumulation of such errors may ultimately lead to cell death. Thus, a change in the expression level of **FANCM** may result in pregnancy loss. While many of these identified signature genes already have a background for the respective co-morbidity that we are studying here, some of them still are bereft of literature. Thus, they could further be studied to get a better insight into their role in TS.

As of now, in the turner population, the relationship between T2DM and RM has not been thoroughly identified. In the context of interacting genes coding for disease-causing putative proteins, we found that FANCM and its interacting partner participate in the DNA repair pathway and Fanconi anemia pathways (also involved in DNA repair). The Fanconi anemia pathway repairs DNA interstrand crosslinks in the genome. Interestingly, one of the interacting partners of FANCM (signature gene of RM) is FANCL which is the interacting partner of GPRC5B (signature gene of T2DM) in the TS PPI network. The association between DM (Type 1 and 2) and DNA damage is well recognized^42,43^ but very little is known about DNA damage in pregnancy, particularly when pregnancy is complicated by pre-gestational or gestational diabetes mellitus^44^.

By analyzing the proportions of chromosomes involved, it was observed that it is not just the genomic imbalance of the genes lying on the deleted pseudoautosomal regions of the X chromosome, but the additive influences of the associated genes located on autosomal chromosomes as well, that may be responsible for TS phenotype. The developmental transition from fetus to adult requires gene expression changes that help in this transition. When a fetus carries a partial or a total loss of the X chromosome, there is a disturbance in the gene expression as compared to a normal fetus. We identified a list of genes that changed their expression pattern in transition from TS fetus to TS adult. It is expected that when these genes are activated in adult or fetal tissues in an altered fashion, this may hinder the developmental processes. While it cannot be explained what causes these fluctuations and how these fluctuations affect the phenotype of Turner patient, this would shed light on a new aspect which requires future insights.

Though the TS network shows a weak hierarchy, it exhibits system-level organization involving modules/communities which are interrelated. Being hierarchical means that there is no significance of individual gene activities rather they work in synchronization to regulate the network. The leading hubs (high degree genes) present in the network play important functions by integrating the lower degree nodes for organizing and regulating activities like inter and intra crosstalk among various other essential genes and thereby maintains network stability and adjusts its signal processing. Of all the seed genes, we identified nine key regulators, namely LCP2, PTPN22, CCL22, CXCL5, S1PR4, POU2F1, FAM20A, ENAM, and EFNB3 which influences network/module regulation and maintains the network stability working as its backbone till the last level. These key regulators could be a possible therapeutic target gene for TS. Earlier it has already been established that PTPN22 polymorphism is related to autoimmune disease risk in patients with Turner syndrome^45^. Surprisingly, only two of the key regulators i.e., LCP2 and EFNB3 were among the top 50 high degree hubs. Thus, it is not necessary for the leading hubs to be the key regulators in the network, their popularity can change randomly at different levels of an organization. All the KRs maintained a low profile/popularity thereby regulating the network till the last level of organization. The regulating ability of KRs is more significant at the deeper level of the organization. The network exhibits fractal nature because its topological properties obey a power law, and a strong LCP is also maintained which means that networks are dynamic and heterogeneous. This indicates that the network maintains self-organization and is compact and has effectual processing information.

Essential evolutionary proteins being more conserved are expected to frequently interact with each other. Based on this fact, we found 10 important interologs (evolutionarily conserved protein–protein interactions) involving the interaction of seed/key genes and their neighbor in TS and 3 motifs in 7 different organisms from lower to a higher level. We considered these non-human systems because the cases of monosomy X have already been reported in these organisms^46–51^. However, in the case of TS, no animal models have been generated that exactly models it. Therefore, as there is a great lack of non-human models to study TS, our findings through orthologous study update current models of TS, thereby giving a bit clear picture of the interologs which are functional in other lower to a higher level of animal models.

Taken together, these results offer few key regulators and essential genes that may act as therapeutic targets for TS in the future. Although this study uncovers many aspects of TS, there are many limitations such as limited sample size and heterogeneity of the datasets used in this study. As no two turner patients are identical, every Turner is unique with respect to its genotype-phenotype. The currently available datasets do not allow a more elaborated study at this moment. Thus, a larger sample size would provide a more elaborate result. Another limitation of our study is that we did not use the adjusted p-value for multiple comparisons, rather we used the p-value cut-off < 0.05 (nominal testing) to select maximum number of DEGs of Turner Syndrome. Also, the co-morbidities studied here are heterogeneous in nature and there may be other factors too that may contribute to their occurrence. Despite these biological limitations, our computational approach and the results offer a comprehensive picture, elucidating the KRs of TS using network biology and demonstrating the importance of animal models in TS, which helps explore and understand different aspects of this syndrome.

## Methodology

### Retrieval of microarray data (TS, T2DM, and RM)

Widely accessible gene expression datasets related to Turner Syndrome (TS), Type 2 Diabetes Mellitus (T2DM), and Recurrent Miscarriage (RM) of *Homo sapiens* were obtained from the Gene Expression Omnibus (GEO) database of NCBI^52^. Studies evaluated on Affymetrix human gene expression dataset containing samples from both normal and diseased tissues of women were taken. For TS, we retrieved GSE46687 deposited by Bondy et al*.* and GSE58435^53^. For T2DM, we retrieved GSE23343^54^ and GSE25724^55^. And in the case of RM, we retrieved GSE22490^56^ and GSE26787^57^. Before finding the differentially expressed genes, these datasets were pre-processed to remove the noise of obscure variations of these data to make them cross-comparable. Normalization is a key step in the process of pre-processing to remove such variations in the data. In this study, we used MAS5 algorithm^58^ which is sensitive and selective for identifying differentially expressed genes. MAS5.0 combines the signals from the multiple Perfect-Match (PM) and Mismatch (MM) probes that target each transcript into a single value that sensitively and accurately represents its concentration by calculating a robust average of the (logged) PM-MM values. After normalizing the datasets, the differentially expressed genes were identified through GEO2r by filtering the genes based on Log_2_FC and P-value.

### Differentially expressed genes of TS, T2DM, and RM

We performed the comparison on normal vs. disease samples in each GEO dataset to identify differentially expressed genes (DEGs). We identified these DEGs through the online program, GEO2R^59^, which is based on limma R package^60^. We chose the “P value < 0.05” and “|logFC|≥ 0.5” as the primary cut-off criteria to interpret the results. In each category (i.e., TS, T2DM, and RM), only those DEGs that satisfied the cut-off criteria in both its datasets were considered as the significant DEGs. To obtain the list of overlapping DEGs, we used Venny 2.1.0, an online tool that can calculate the intersection(s) of listed elements. The enriched functions and biological pathways involved with these DEGs were identified using DAVID (The Database for Annotation, Visualization and Integrated Discovery) online server^61^ and REACTOME pathway browser^62^.

### Protein–protein interaction network construction of TS and their topological properties

To analyze the interactive associations among the DEGs at the protein level, genes obtained from the TS were mapped on protein–protein interaction (PPI) data using STRING database^63^ to construct the TS PPI network with a medium confidence score with 400 nodes in 1^st^ shell interactors and 100 nodes in 2^nd^ shell interactors so that more number of seed genes make into the network. The Search Tool for the Retrieval of Interacting Genes/Proteins (STRING) database aims to integrate comprehensive PPI data available from different databases for a large number of organisms from published literature with experimental information. The network was visualized in Cytoscape 3.4^64^. These DEGs are said to be the seed genes. The structural properties of complex networks are characterized through the behaviors of their topological parameters. The topological properties of the TS network were calculated by Network Analyzer and CytoNCA^65^ in Cytoscape. The topological properties analyzed in the present study are described below.

#### Degree distribution

In a PPI network, the number of contacts a node/protein has with other nodes/proteins are said to be its degree and the probability distribution of these degrees over the entire network is the degree distribution. The networks whose degree distributions approximately follow a power law: P(k) ~ k^−γ^, where γ is a constant are termed as scale-free networks and appear linear in a log–log plot. Depending on the value of γ the networks are said to be hierarchical which further specifies the importance of hubs or modules in the network^66^. The concept of a scale-free network is used to separate biological networks from random networks, which follow a Poisson distribution. For a PPI network defined by a graph G = (N, E), where N and E are the number of nodes and edges respectively, the probability of degree distribution (P(k)) is the ratio of the number of nodes with degree k to the network size.

3 $$ P\left( k \right) = \frac{{n_{k} }}{N} $$

where n_k_ is the number of nodes having degree k and N is the total number of nodes in the network. P(k) indicates the importance of hubs or modules in the network.

#### Neighborhood connectivity

In a PPI network, when a node/protein ‘n’ forms an association with its neighbor nodes/proteins, the average number of neighbors of all the nearest neighbors of this node ‘n’ is said to be its Neighborhood connectivity^67^. In the network (C_N_(k)) Neighborhood connectivity is given by,

4 $$ C_{N} \left( k \right) = \mathop \sum \limits_{q} qP\left( \frac{q}{k} \right) $$

where, P($\frac{q}{k}$) is the conditional probability that a connection belonging to a node with connectivity k points to a node with connectivity q. The positive power dependence of C_N_(k) indicates assortivity in the network topology.

#### Clustering co-efficient

In a PPI network, the clustering coefficient represents the measure and strength of how connected the neighbors of a given node are in that network. It measures the tendency of a node to form a cluster. Identifying these modules/communities is significant because they can ultimately reflect functional modules and protein complexes. When applied to an entire network, the clustering coefficient is its average over all the nodes in the network. It is calculated by the ratio of the number of its nearest neighborhood edges e_i_ to the total likely number of edges of degree k_i_. For an undirected network, the clustering co-efficient (C(k_i_)) of ith node can be calculated by,

5 $$ C\left( {k_{i} } \right) = \frac{{2e_{i} }}{{k_{i} \left( {k_{i} - 1} \right)}} $$

#### Betweenness centrality

Betweenness centrality (C_B_) of a node in a PPI network measures the degree of information flow in the network. It is the capacity of a protein/node to monitor communication between other proteins/nodes in a network^68,69^. If d_ij_ (v) indicates the number of geodesic paths from node i to node j passing through node v, and dij indicates the number of geodesic paths from node i to j, then betweenness centrality (C_B_(v)) of a node v can be calculated by,

6 $$ C_{B} \left( v \right) = \mathop \sum \limits_{i,j,i \ne j \ne k} \frac{{d_{ij} \left( v \right)}}{{d_{ij} }} $$

#### Closeness centrality

Closeness centrality (C_C_) measures how fast the flow of information is from a node to other nodes reachable from it in the network^70^. Therefore, it shows how close a node ‘n’ is to all other nodes in a network. C_C_ of a node i is the reciprocal of the mean geodesic distance between the node and all other nodes connected to it in the network and is given by,

7 $$ C_{C} \left( i \right) = \frac{n}{{\mathop \sum \nolimits_{j} d_{ij} }} $$

where d_ij_ represents the geodesic path length from nodes i to j, and n is the total number of vertices in the graph reachable from node i.

#### Eigenvector centrality

In a PPI network, eigenvector centrality is a tendency of a node to enable the information to spread in a network. It measures the significance of a node while considering the significance of its neighbors. The main idea behind eigenvector centrality is that connections from significant nodes are more important than connections from unimportant nodes. Eigenvector centrality of a node i (C_E_(i)) in a network is proportional to the sum of i’s neighbor centralities^71^, and it is given by,

8 $$ C_{E} \left( i \right) = \frac{1}{\lambda }\mathop \sum \limits_{j = nn\left( i \right)} v_{j} $$

where nn(i) indicates the nearest neighbors of nodes i in the network. λ is eigen value of the eigenvector v_i_ is given by, Av_i_ = λv_i_ where A is the adjacency matrix of the network (graph).

### Validation of the biological significance of TS network

It is said that scale-free networks are robust against random removals of nodes because most nodes are poorly connected, and they play relatively unimportant roles in organizing the global network structure. The TS PPI network constructed in this study follows scale-free features and consists of 775 nodes and 20,357 edges. To check whether a similar network would arise if a random set of genes of the same size as the TS DEG-set are used as "seeds," or whether the topology of the resulting network would be obtained by randomly sampling the STRING-db, we constructed the null random networks of the same size and did the comparative analysis. In our study, we constructed the random networks through Network Randomizer App^72^ in Cytoscape considering two different scenarios:

- randomization of the constructed TS network by **Preserving the Degree**.

Through Network Randomizer, we first randomized the TS network by preserving the degree of each node. The degree preserving shuffling algorithm permits to randomize the current network considering the degree of each node. This means that in the randomized network, a node will have the same number of neighbors, but they can be different.

- construction of a random network of the same size through the **Erdős and Rényi algorithm**.

Next, we generated a random network with 775 nodes and 20,357 edges using an Erdös–Rényi model^73^ in which for each pair of nodes, a link was inserted with independent probability. We used the G (n, M) model to construct the uniform random graph where n is the number of nodes and M is the number of edges.

We then did the comparative analysis of these random networks with the main TS network.

### Community detection: leading eigen-vector method and tracing of the genes

The constructed PPI network is divided into discrete layers of hierarchy. Each layer or tier describes its activity which altogether defines the modular nature, properties, and organizing principle of the hierarchical network. We used the Leading Eigen Vector method (LEV)^74,75^ to detect the communities of the network in R from package ‘igraph’^76^ in this study. The LEV method calculates the eigen value for each connection, demonstrating the importance of each connection, not nodes. The modules were detected from the main network, then from the sub-modules of the modules, at each level of hierarchy to finally obtain the motif. The seed genes were then traced at each level of organization in various modules/sub-modules obtained from clustering. The genes reaching the motif level (last level) are the main drivers of the TS network that helps in its regulation. We consider these genes as the most significant and influential ones within the network and call them the key regulators of the network. The Probability $P_{x} \left( {y^{l} } \right)$ of KR was then calculated to recognise the regulating ability of each of these KRs in the TS network,

9 $$ P_{x} \left( {y^{\left[ l \right]} } \right) = \frac{{y^{\left[ l \right]} }}{{E^{\left[ l \right]} }} $$

where x is the number of edges *y*^*[l]*^ at level l and *E*^*[l]*^ is the total number of edges of the network/ modules/sub-modules.

### Distribution of energy in the network: Hamiltonian energy calculation

Each level of the network is organized and maintained due to a certain level of energy. This energy is measured at each level using Hamiltonian Energy (HE) within the formalism of Constant Potts Model^77,78^. HE calculates the energy distribution at the global level as well as at the modular level. HE of a network or module or sub-module can be calculated by,

10 $$ H^{\left[ c \right]} = - \mathop \sum \limits_{c} [e_{c} - {\upgamma }n_{c }^{2} ] $$

where e_c_ and n_c_ are the number of edges and nodes in a community ‘c’ and γ is the resolution parameter acting as an edge density threshold which is set to be 0.5.

### Local-community-paradigm (LCP) approach: compactness of the network

LCP-Decomposition Plot (LCP-DP) is a method to characterize the topological self-organization of a network as a local-community-paradigm (LCP). It is used to study the effect of LCP on network topology. It is a function of the common neighbors (CN) index and local community links (LCL) of each pair of interacting nodes in the network. This approach gives information on the number, size, and compactness of the communities in a network^79^. The CN index between two nodes x and y is the measure of overlapping between their sets of first node neighbors S(x) and S(y) given by, $CN = S\left( x \right) \cap S\left( y \right)$. A significant amount of overlapping indicates a possible likelihood of interaction of these two nodes. Therefore, an increase in CN represents the increase in compactness of the network representing its faster information processing abilities. Further, the LCLs between the two nodes x and y, whose upper bound is defined by, $\max \left( {LCL} \right) = \frac{1}{2}CN\left( {CN - 1} \right)$, is the number of internal links which is strongly inter-linked in local-community (LC). These two nodes most probably link together if CN of these two nodes is members of LC^79^. LCP-DP has a linear dependence between CN and $\sqrt {LCL}$.

The LCP correlation (LCP-corr) is the Pearson correlation co-efficient between the variables CN and LCL defined by

11 $$ LCP - corr = \frac{{cov\left( {CN,LCL} \right)}}{{{\upsigma }_{CN} {\upsigma }_{LCL} }} $$

with CN > 1, where cov(CN, LCL) is the covariance between CN and LCL, σ_CN_ and σ_LCL_ are standard deviations of CN and LCL, respectively.

### Status of essential interactions of TS in non-human systems by integrating the Orthology with PPI

A positive relationship exists between essentialities (essential proteins) and topological properties (centralities) of the proteins in PPI networks. Therefore, a series of network topological features based on centrality measures have been used to recognize essential proteins. The properties considered in this study are Degree Distribution, Betweenness Centrality, Closeness Centrality, and Eigenvector Centrality. The proteins of the TS network were graded in terms of these topological properties (top 100 in each category). These ranking scores were then used to predict whether a protein is essential or not. Further, the interacting partners of the seed genes/proteins were also identified in the TS network. The interacting partners of seed genes/proteins that were common to all these four properties were considered essential.

In this study, we predicted the essential proteins in the TS network by integrating the orthology with the PPI network of TS. The essential proteins are more evolutionarily conserved than non-essential proteins and they frequently interact with each other. To identify the conserved interaction of the TS network, the selected interactions were analyzed in 7 different species namely, *Mus musculus* (mouse), *Rattus norvegicus* (Rat), *Ovis aries* (sheep), *Felis catus* (domestic cat), *Macaca mulatta* (rhesus macaque), *Gorilla Gorilla* (Gorilla), and *Homo sapiens* (human) (lower to higher-level organisms). For this, information on orthologs of selected proteins was taken from Version 8 of the InParanoid database (an ortholog database)^80^ and Orthodb v10.1^81^. Then the networks of the selected organisms were constructed considering these essential interacting proteins as seed genes for the analysis of the conserved interactions from the STRING database with a 0.7 confidence score to get high confidence interactions.

## Supplementary Information

Supplementary Information 1.

Supplementary Information 2.
