# Supplementary material for: Network-based analysis of key regulatory genes implicated in Type 2 Diabetes Mellitus and Recurrent Miscarriages in Turner Syndrome
Source: Sci Rep. 2021 May 21;11:10662. doi: 10.1038/s41598-021-90171-0 (PMC8140125; doi:10.1038/s41598-021-90171-0)

# Network-based analysis of key regulatory genes implicated in Type 2 Diabetes Mellitus and Recurrent Miscarriages in Turner Syndrome

Anam Farooqui[1], Alaa Alhazmi[2], Shafiul Haque[3], Naaila Tamkeen[4], Mahboubeh Mehmankhah[1], Safia Tazyeen[1], Sher Ali[5] and Romana Ishrat[1*]

[1]Centre for Interdisciplinary Research in Basic Science, Jamia Millia Islamia, New Delhi-110025, India
[2]Medical Laboratory Technology Department, Jazan University, Jazan-45142, Saudi Arabia
[3]Research and Scientific Studies Unit, College of Nursing and Allied Health Sciences, Jazan University, Jazan-45142, Saudi Arabia
[4]Department of Biosciences, Jamia Millia Islamia, New Delhi-110025, India
[5]Department of Life Sciences, Sharda University, Greater Noida -201310, India

*Corresponding Author:
Dr Romana Ishrat (Associate Professor)
Centre for Interdisciplinary Research in Basic Sciences,
Jamia Millia Islamia, New Delhi-110025, India.
Email address – romana05@gmail.com

**Fig S1. The Boxplots of (A) GSE46687 and (B) GSE58435 microarray dataset of Turner Syndrome (TS) after normalisation**

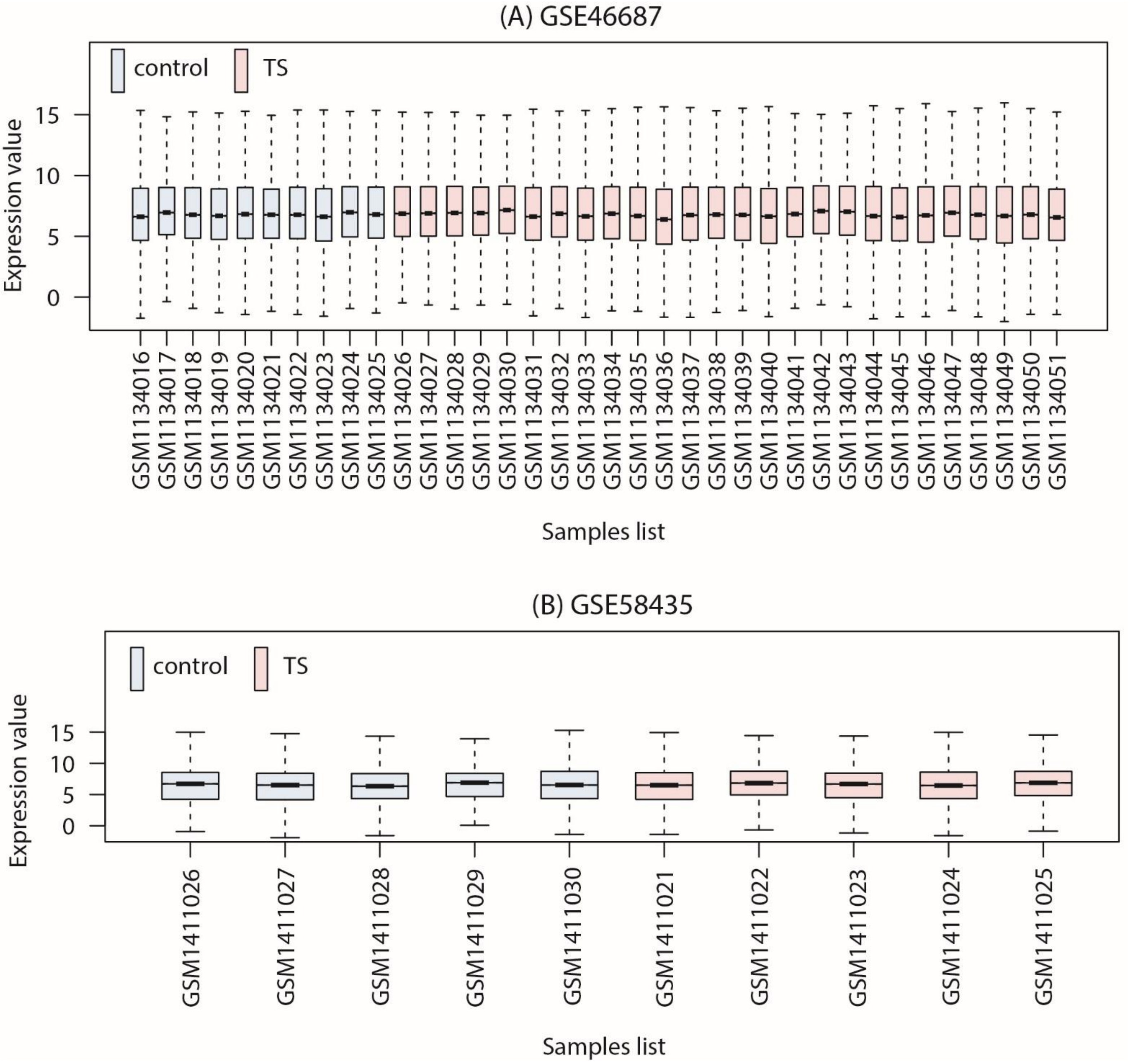

**Fig S2. The Boxplots of (A) GSE23343 and (B) GSE25724 microarray dataset of Type 2 Diabetes Mellitus (T2DM) after normalisation**

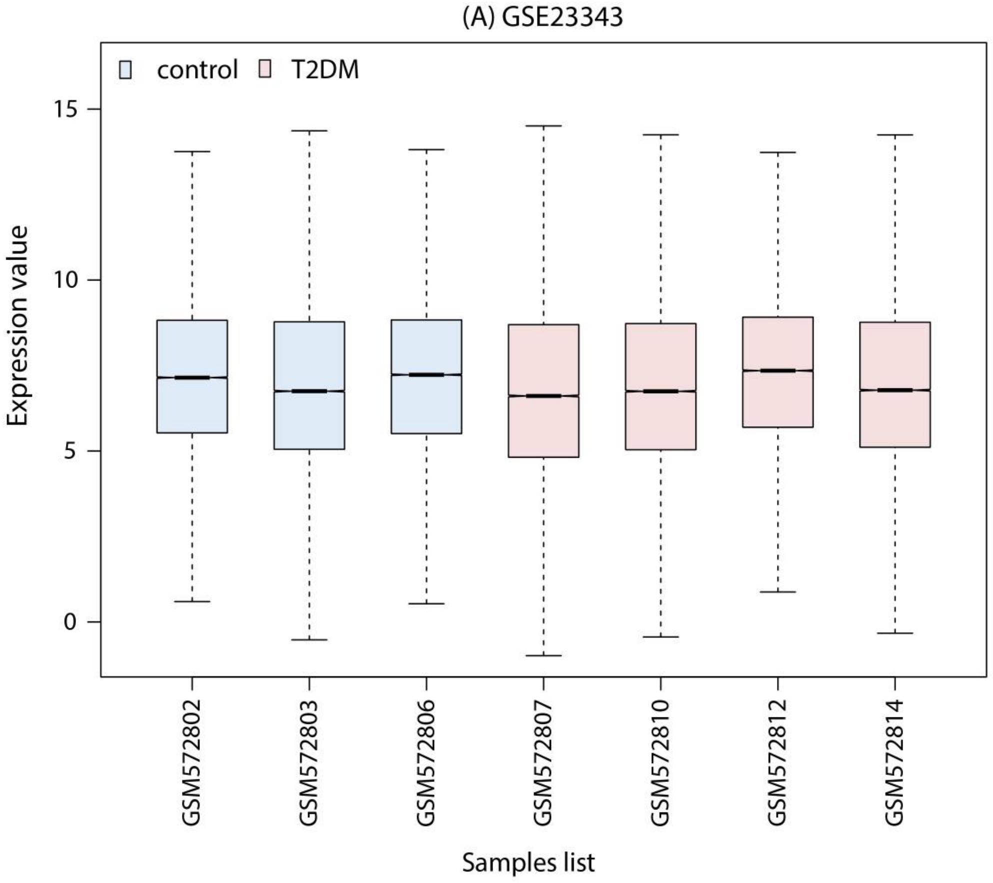


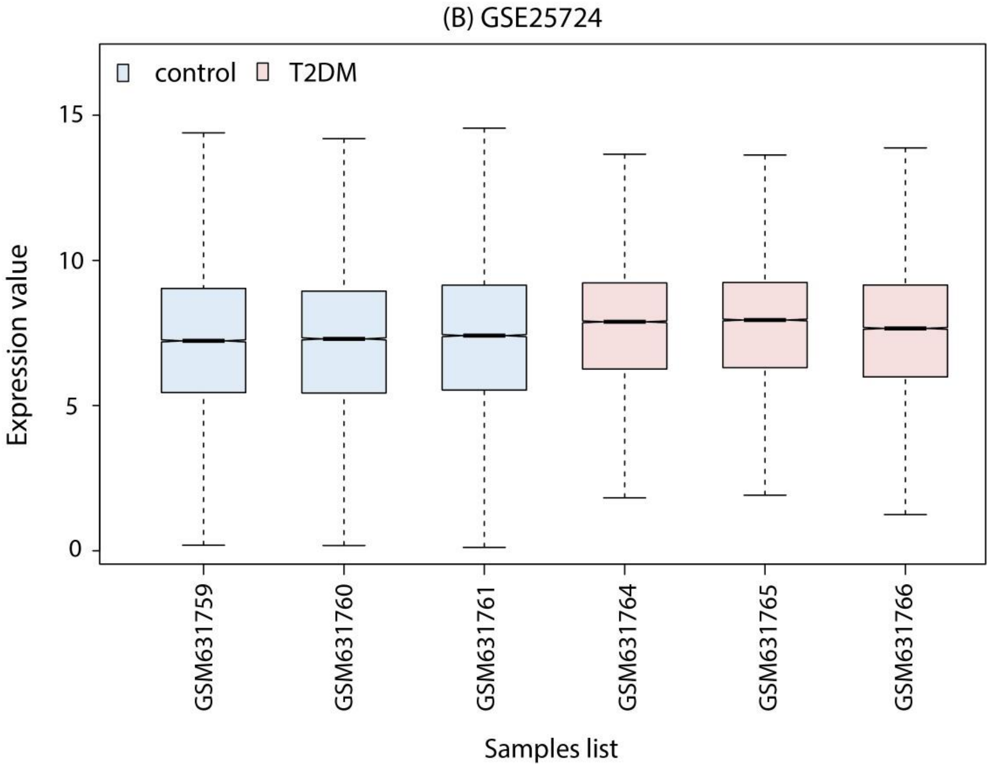

**Fig S3. The Boxplots of (A) GSE22490 and (B) GSE26787 microarray dataset of Recurrent Miscarriage (RM) after normalisation**

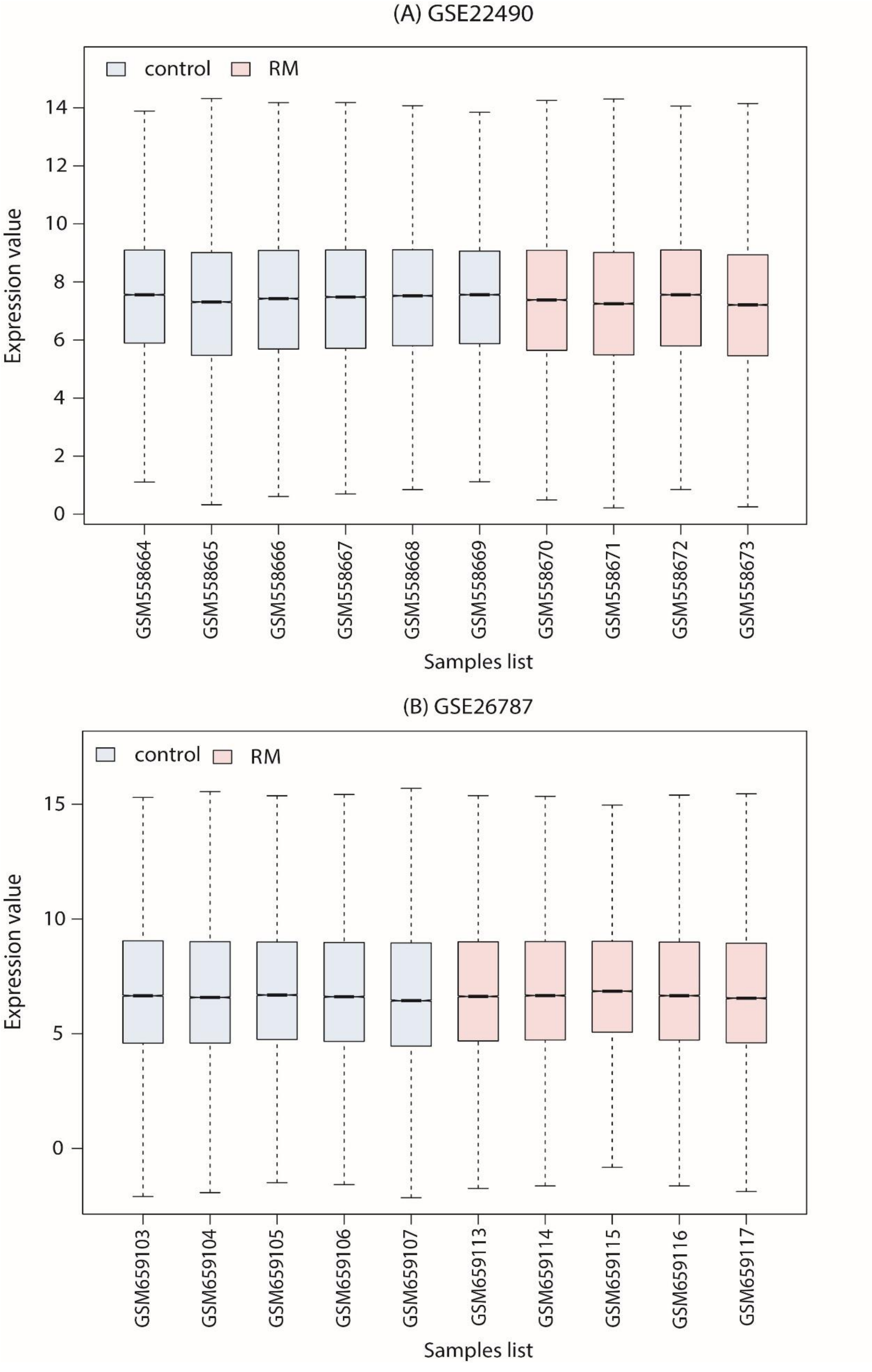

**Fig S4. (A) Common DEGs of TS (B) Common DEGs of T2DM (C) Common DEGs of RM**

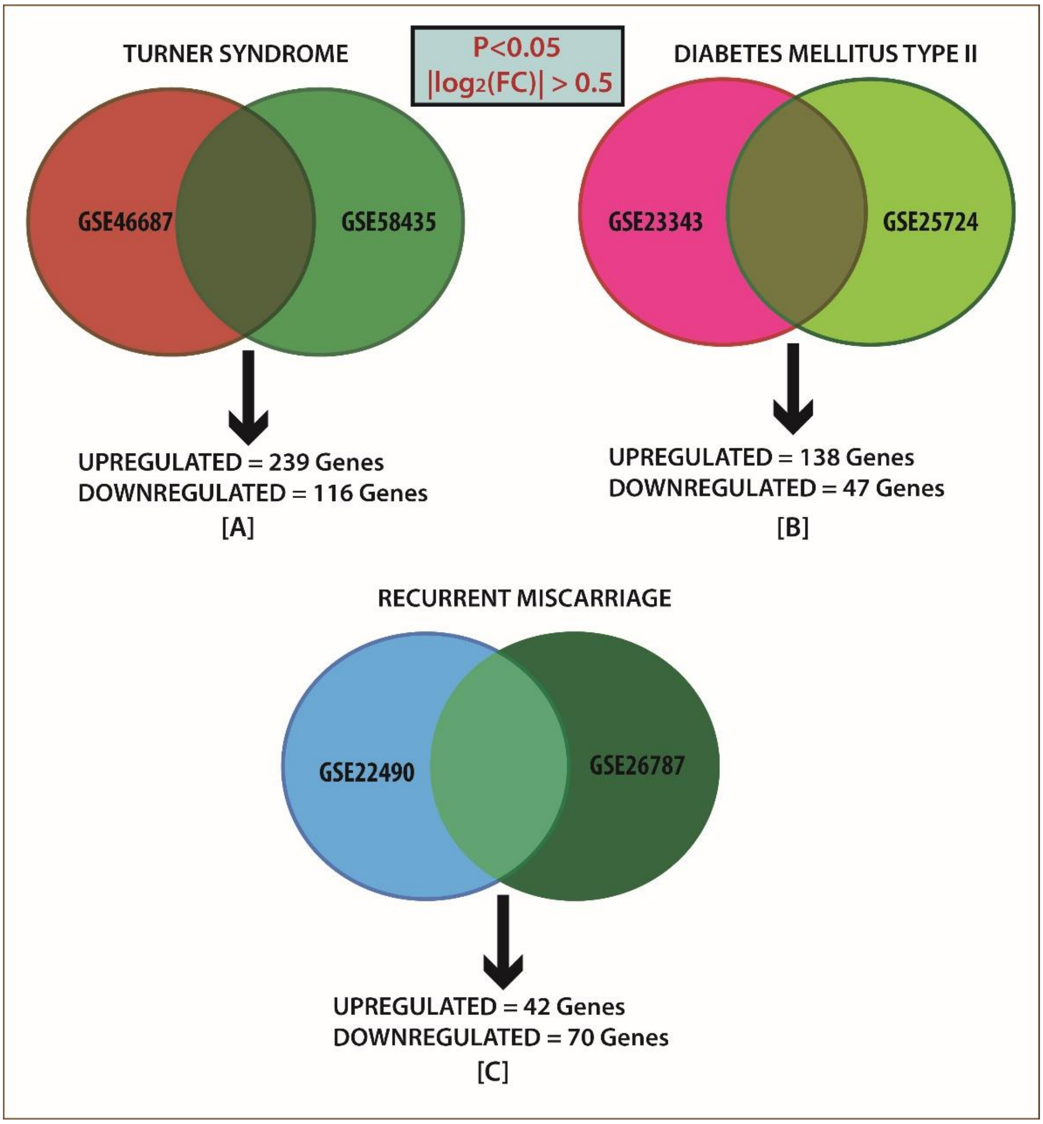

**Fig S5. Overview of the results of pathway analysis of DEGs and signature genes along with their interacting partners.** Reactome pathways are arranged in a hierarchy. The center of each of the circular "bursts" is the root of one top-level pathway, for example "DNA Repair". Each step away from the center represents the next level lower in the pathway hierarchy. The color code denotes over-representation of that pathway in dataset. Light grey signifies pathways which are not significantly over-represented. The pathways that are actively involved are highlighted in yellow. DNA repair and Fanconi Anaemia pathway are highlighted in red as they are involved with signature genes and their interacting partner.

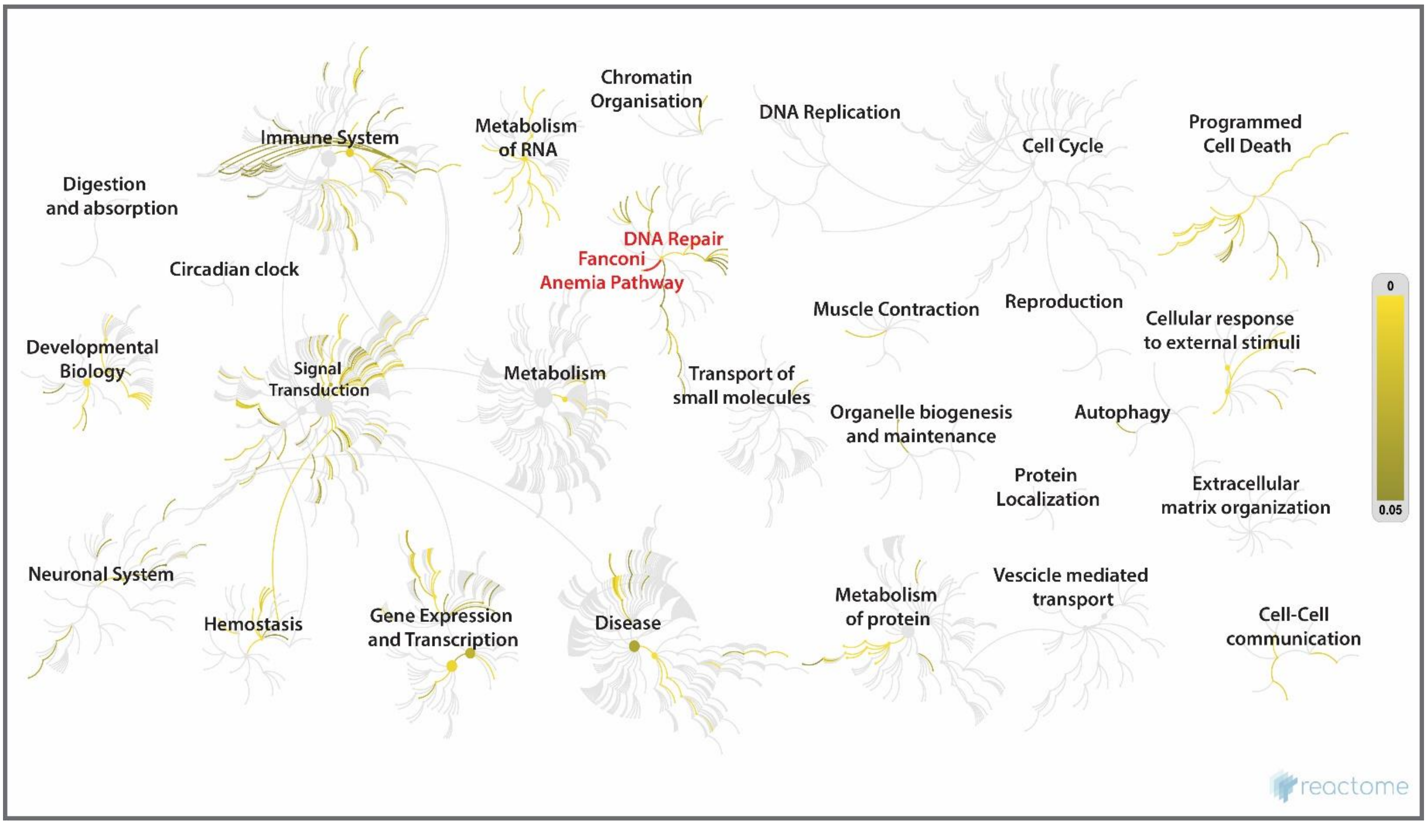

**Fig S6.** The behaviours of degree distributions (P(k)), clustering co-efficient (C(k)), neighborhood connectivity ($C_N(k)$), betweenness ($C_B(k)$), closeness ($C_C(k)$) and eigen-vector ($C_E(k)$) measurements as a function of degree k for **[a]** TS network **[b]** randomized TS network by **Preserving the Degree [c]** random network of same size through **Erdős and Rényi algorithm**

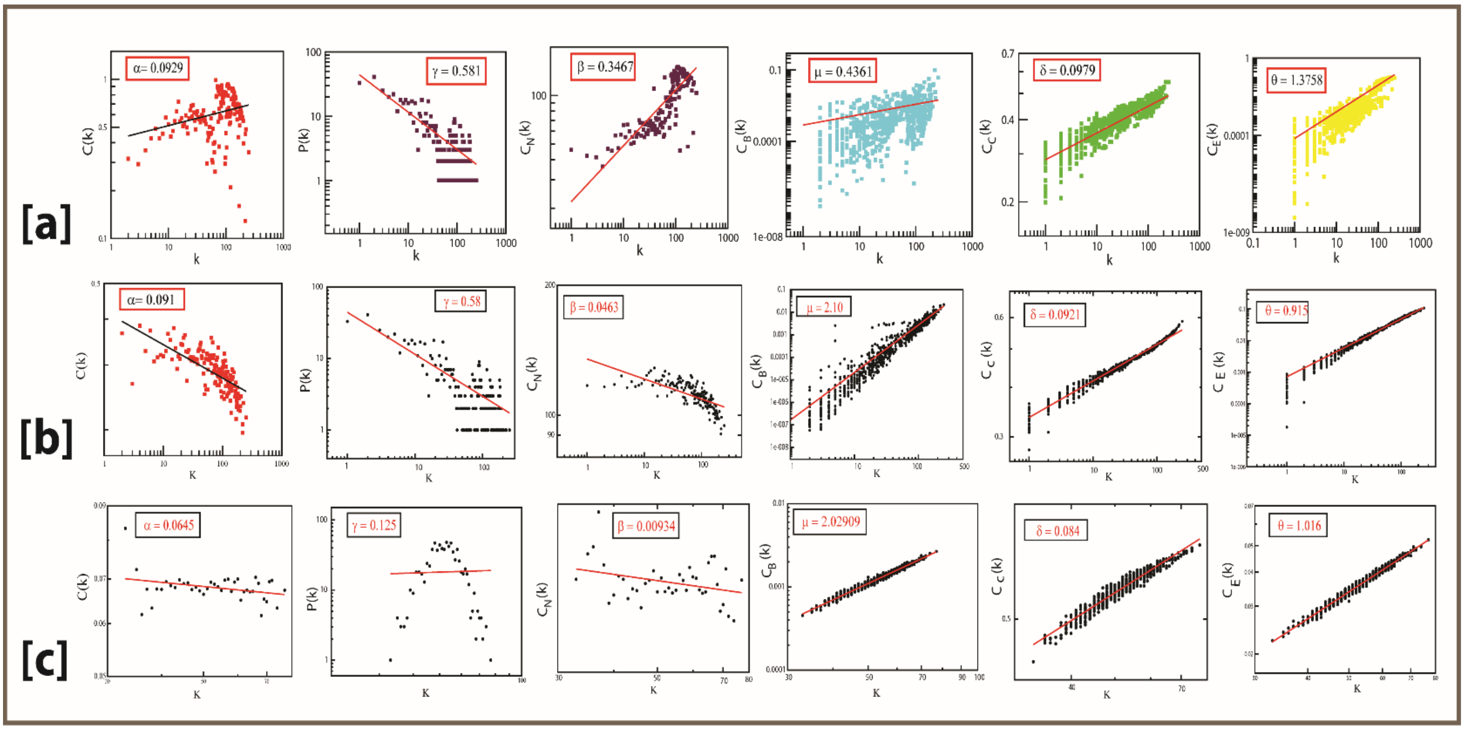

Supplement: Supplementary file 1 — Supplementary Information 1. [file 41598_2021_90171_MOESM1_ESM.pdf]
